# The Clinical Utility of Genetic Testing in the Diagnosis and Management of Adults with Chronic Kidney Disease

**DOI:** 10.1681/ASN.0000000000000249

**Published:** 2023-10-05

**Authors:** Neera K. Dahl, Michelle S. Bloom, Fouad T. Chebib, Dinah Clark, Maggie Westemeyer, Sara Jandeska, Zhiji Zhang, Hila Milo-Rasouly, Victoria Kolupaeva, Maddalena Marasa, Varshasb Broumand, Richard A. Fatica, Dominic S. Raj, Zachary P. Demko, Kyle Marshall, Sumit Punj, Hossein Tabriziani, Sangeeta Bhorade, Ali G. Gharavi

**Affiliations:** 1 Division of Nephrology and Hypertension, Mayo Clinic, Rochester, Minnesota; 2 Natera, Inc., Austin, Texas; 3 Division of Nephrology and Hypertension, Mayo Clinic, Jacksonville, Florida; 4 Division of Nephrology, Department of Medicine, Columbia University College of Physicians and Surgeons, New York, New York; 5 South Texas Renal Care Group, San Antonio, Texas; 6 Department of Kidney Medicine, Cleveland Clinic, Cleveland, Ohio; 7 Division of Kidney Diseases and Hypertension, George Washington University School of Medicine and Health Sciences, Washington, DC

**Keywords:** CKD, clinical trial, genetic kidney disease

## Abstract

**Significance Statement:**

Accurate diagnosis of a patient's underlying cause of CKD can influence management and ultimately overall health. The single-arm, interventional, prospective Renasight Clinical Application, Review, and Evaluation study assessed the utility of genetic testing with a 385 gene kidney disease panel on the diagnosis and management of 1623 patients with CKD. Among 20.8% of patients who had positive genetic findings, half resulted in a new or reclassified diagnosis. In addition, a change in management because of genetic testing was reported for 90.7% of patients with positive findings, including treatment changes in 32.9%. These findings demonstrate that genetic testing has a significant effect on both CKD diagnosis and management.

**Background:**

Genetic testing in CKD has recently been shown to have diagnostic utility with many predicted implications for clinical management, but its effect on management has not been prospectively evaluated.

**Methods:**

Renasight Clinical Application, Review, and Evaluation RenaCARE (ClinicalTrials.gov NCT05846113) is a single-arm, interventional, prospective, multicenter study that evaluated the utility of genetic testing with a broad, 385 gene panel (the Renasight^TM^ test) on the diagnosis and management of adult patients with CKD recruited from 31 US-based community and academic medical centers. Patient medical history and clinical CKD diagnosis were collected at enrollment. Physician responses to questionnaires regarding patient disease categorization and management were collected before genetic testing and 1 month after the return of test results. Changes in CKD diagnosis and management after genetic testing were assessed.

**Results:**

Of 1623 patients with CKD in 13 predefined clinical disease categories (ages, 18–96; median, 55 years), 20.8% (*n*=338) had positive genetic findings spanning 54 genes. Positive genetic findings provided a new diagnosis or reclassified a prior diagnosis in 48.8% of those patients. Physicians reported that genetic results altered the management of 90.7% of patients with a positive genetic finding, including changes in treatment plan, which were reported in 32.9% of these patients.

**Conclusions:**

Genetic testing with a CKD-focused 385 gene panel substantially refined clinical diagnoses and had widespread implications for clinical management, including appropriate treatment strategies. These data support the utility of broader integration of panels of genetic tests into the clinical care paradigm for patients with CKD.

**Clinical Trial registry name and registration number:**

ClinicalTrials.gov, NCT05846113.

## Visual Abstract

**Figure:**
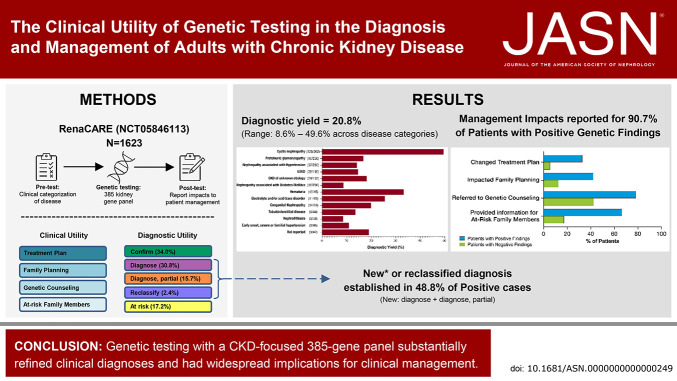

## Introduction

CKD affects 10% of the global population and was the 10th leading cause of death in 2020.^1^ CKD represents a significant economic burden on the health care system. In 2020, 23.5% of Medicare spending, $85.4 billion, involved in the management and treatment of CKD.^2^

CKD's significant morbidity and mortality may, in part, be due to underdiagnosis and misdiagnosis, resulting in poor clinical outcomes. The most commonly reported causes of CKD in adults are diabetes and hypertension; however, these are often comorbidities and not primary causes.^3^ In addition, 18% of ESKD cases have an unknown etiology.^2^ As such, the true proportion of CKD cases in which the underlying cause remains unrecognized is likely underestimated.

The implementation of new sequencing technologies in patients with CKD has expanded understanding of the underlying genetic causes of CKD pathologies. Disease-causing variants have been described in over 600 nephropathy-associated genes, and monogenic etiologies have been identified in up to 30% of adults with CKD and in 12%–56% of patients with CKD/ESKD of unknown etiology.^4–10^ The high rate of genetic findings in CKD suggests that there may be a greater role for genetic testing in CKD management, similar to the more widespread adoption seen in oncology where the overall prevalence of heritable causes of cancer is lower.^6,11^ A number of studies have demonstrated that multiple barriers, including physician education around genetics, are hindering the widespread use of genetic testing in nephrology.^12,13^ Addressing these gaps could serve to help establish genetic testing as a routine part of clinical workup in nephrology.

Identification of a monogenic cause of CKD can have profound effects on the management of the increasing numbers of CKD-related diseases with targeted therapies. It can identify appropriate treatments to slow disease progression, inform disease prognosis, enable evaluation for extrarenal manifestations, and quantify disease recurrence risk after kidney transplant. Moreover, it also has implications for family members and can inform testing of at-risk relatives, reproductive decision making, and screening of potential living-related kidney donors. These far-reaching implications of genetic testing were recently recognized by a report on kidney genetics from Kidney Disease Improving Global Outcomes, which stated that genetic testing should be integrated into standard CKD patient evaluation.^14^

Groopman *et al.* reported that 9.3% of patients with CKD had monogenic etiologies by whole-exome sequencing, of which 89% had putative implications for clinical management.^6^ Initial analysis of patients tested using a commercially available 385 kidney disease-gene panel reported a diagnostic yield of 21.1% spanning both common and rare diseases, demonstrating the clinical applicability of genetic testing in a real-world setting.^15^ However, there remains a need to prospectively evaluate the effects on patient management after genetic testing. In this study, we present an interim analysis of the primary end points of the Renasight Clinical Application, Review, and Evaluation (RenaCARE) study that sought to expand on these previous findings to evaluate the real-world diagnostic and clinical utility of broad panel genetic testing in patients with CKD.

## Methods

### Study Design and Participants

RenaCARE (ClinicalTrials.gov NCT05846113) is an open-label, interventional, single-arm, unblinded prospective, multicenter study designed to evaluate the diagnostic and clinical utility of genetic testing with a next-generation sequencing (NGS)–based 385 kidney gene panel (the Renasight^TM^ test, Natera, Inc., Austin, TX) on patients with CKD. Physicians from 31 US-based academic or community practices (Supplemental Figure 1 and Supplemental Table 1) evaluated adult patients (older than 18 years) with a CKD diagnosis for inclusion in this study. Eligible patients included those with a CKD diagnosis and/or fell into one of 13 clinical kidney disease categories specified in the inclusion criteria (Supplemental Table 2).

All study participants signed a local institutional review board-approved informed consent before enrollment. Genetic testing with the Renasight test was provided to all patients on enrollment in this study. Blood or saliva swabs were collected for genetic testing at the time of in-person enrollment or shipped to the sequencing laboratory for patients who were consented remotely. Test results were provided to participating physicians and used for clinical decision making at their discretion.

Sample size calculations for this study were based on the observed positive test rate of 9.3%^6^ and yielded a minimum of 811 patients to be enrolled to estimate the frequency of positive test results to within ±0.02. To ensure representation of all clinical disease categories, enrollment was capped for categories that exceeded 10% of the total cohort (per the study protocol). In accordance with this, enrollment of patients categorized with cystic nephropathies, nephropathies associated with hypertension, or nephropathies associated with diabetes mellitus was capped.

Demographic information (age, race, ethnicity, and sex at birth), clinical data, and medical history (including histopathologic diagnosis, when present, imaging and chemistry) were documented at enrollment by clinical research coordinators (pretest) (Supplemental Table 3). For each patient, ethnicity was reported through defined selections as follows: Hispanic or Latino, not Hispanic or Latino, or unknown. Race was reported through the selection of any of the following categories: American Indian or Alaska native, Asian, Black or African American, White, other (write in), unknown, or not reported. Referring physicians were asked to provide information about kidney disease categorization/diagnosis and the management of each patient through questionnaires at enrollment (pretest) and 1 month after the return of genetic test results (post-test) (Supplemental Tables 4 and 5). Kidney disease categories represent broad clinical presentations to ensure capturing all potential etiologies. In certain cases where a patient's presentation may have fit into more than one category, physicians selected the most appropriate or primary presentation. The primary outcomes of the study included (*1*) test positive prevalence (*i.e.*, diagnostic yield), (*2*) diagnostic and reclassification rates after genetic testing, and (*3*) the effects on patient management after genetic testing, as assessed by the post-test questionnaire. This study was performed in adherence with the Declaration of Helsinki.

### Renasight NGS Panel Sequencing and Data Analysis Variant Interpretation

Genomic material from blood or saliva swabs was processed for library preparation and sequenced by NGS as described previously.^15^ Variants were assessed and classified using a standard operating procedure on the basis of the American College of Medical Genetics and Genomics and Association for Molecular Pathology guidelines for sequence variant interpretation, as previously described.^15,16^ Positive findings included (*1*) a monoallelic pathogenic (P)/likely pathogenic (LP) variant in a gene associated with an autosomal dominant or X-linked disorder, (*2*) biallelic P/LP variants in a gene associated with an autosomal recessive disorder, and/or (*3*) biallelic *APOL1* G1 and/or G2 high-risk alleles. Heterozygous P/LP variants within *COL4A3* and *COL4A4* were considered positive, as were heterozygous P/LP variants in *COL4A5* in female patients.^17^ Identification of high-risk alleles in *APOL1* provides its own counseling and management opportunities.^18^ As the objective of this study was to assess changes to patient management after genetic testing, all positive findings (inducing *APOL1* high-risk genotypes) were considered together for these analyses. All other findings (including patients with a monoallelic P/LP variant for an autosomal recessive disorder [carriers] and/or variants of uncertain significance) or the absence of any of the above findings were classified as negative.

Note: Since the time of testing, an association between the *IFT140* gene and autosomal dominant polycystic kidney disease was established.^19,20^ At the time of testing, heterozygous P/LP variants in *IFT140* were reported as carriers; for the purposes of this analysis, these were treated as negative results (Supplemental Table 6). Similarly, variants of uncertain significance findings for five patients were upgraded to P/LP, resulting in a positive finding, after data collection was completed (Supplemental Table 7). As reported clinical decisions were made before these upgrades, the findings were also treated as negative.

### Clinical CKD Diagnostic Classification with a Positive Genetic Result

For cases with a positive genetic finding, the pretest clinical diagnosis (the clinical categorization and medical history) was reviewed alongside the post-test genetic diagnosis by two independent genetic experts (board-certified genetic counselors [M. Westemeyer and D. Clark]) and/or a board-certified nephrologist [S. Jandeska] and the case was classified into one of the following categories: “confirm” (genetic findings matched pretest clinical diagnosis); “diagnose” (no clinically established cause of CKD; genetic findings explained the full patient phenotype); “diagnose, partial” (no clinically established cause of CKD; genetic findings explained some aspects of the patient phenotype); “reclassify” (genetic findings indicated a different diagnosis than indicated by the physician); and “at-risk finding” (features associated with the genetic findings were not reported by the physician; the risk of development of these features remains; a detailed description of classifications can be found in Supplemental Methods). All diagnostic classifications were then reviewed by an additional domain expert (A.G. Gharavi) with expertise in nephrology and genetics. In cases with no consensus, the reviewers and the domain expert conferred to achieve consistency in classification. The reviewers were conservative in their classifications of cases in which no pretest clinical categorization was provided and/or insufficient clinical information was available. These cases were designated “at-risk” to avoid overestimating the genetic finding effect.

### Statistical Methods

Descriptive statistical analyses were performed to summarize cohort demographics, clinical kidney disease categorizations, and genetic findings prevalence. Diagnostic yield of the genetic test was calculated as the proportion of patients with a positive genetic finding, including *APOL1* high-risk genotypes.

Comparisons between patients with positive and negative genetic findings were analyzed using the two-sided Fisher exact test. Differences in quantitative measures were compared using the Wilcoxon rank-sum test. All tests used an alpha level of 0.05 to determine statistical significance. Adjustments for the type I error rate due to multiple comparisons were made using the false discovery rate method of Benjamini and Hochberg.^21^ *P* values were adjusted when a set of statistical tests could be thought of as a group of related hypotheses (*e.g.*, comparisons within the same table).

## Results

### Cohort Demographics and Clinical History

Between May 2021 and May 2022, a total of 1712 patients were enrolled in the RenaCARE study; 89 patients were excluded due to lack of an evaluable genetic test sample result (Supplemental Figure 2). Among the 1623 patients who underwent genetic testing, the median age was 55 years (range, 18–96) and 51.0% were female. Altogether, 30.4% of patients in the study were Asian American (4.7%), Black/African American (15.5%), or American Indian/Alaska Native/Native Hawaiian/Pacific Islander (1.3%) and 20.6% were of Hispanic ethnicity. Family history of CKD was reported for 36.1% of patients (Table 1). Patients with all stages of CKD (1–5) and ESKD were represented. Among the 13 pretest clinical kidney disease categorizations, the six most common categories accounted for 79.2% of all cases (Table 2).

**Table 1:** Demographics of the Renasight Clinical Application, Review, and Evaluation cohort

Characteristic	Total	Positive Cases^a^	Negative
*N*	1623	338	1285
Age, yr			
Median (range)	55.0 (18.0–96.0)	48.0^b^ (18.0–84.0)	57.0 (18.0–96.0)
Age groups, yr, *n* (%)			
18–39	315 (19.4)	98 (29.0)^b^	217 (16.9)
40–64	913 (56.2)	182 (53.8)	731 (56.9)
≥65	393 (24.2)	57 (16.9)^b^	336 (26.1)
Not reported	2 (0.1)	1 (0.3)	1 (0.1)
Sex at birth, *n* (%)			
Female	827 (51.0)	190 (56.2)	637 (49.6)
Male	794 (48.9)	147 (43.5)	647 (50.4)
Not reported	2 (0.1)	1 (0.3)	1 (0.1)
Race, *n* (%)			
Asian American	77 (4.7)	13 (3.8)	64 (5.0)
Black/African American	251 (15.5)	74 (21.9)^b^	177 (13.8)
American Indian/Alaska Native/Native Hawaiian/Pacific Islander	21 (1.3)	2 (0.6)	19 (1.5)
White	1130 (69.6)	223 (66.0)	907 (70.6)
Other^c^/not reported	158 (9.7)	29 (8.6)	129 (10.0)
Ethnicity, *n* (%)			
Hispanic or Latino	334 (20.6)	58 (17.2)	276 (21.5)
Not Hispanic or Latino	1257 (77.4)	274 (81.1)	983 (76.5)
Unknown	32 (2.0)	6 (1.8)	26 (2.0)
Family history of CKD, *n* (%)			
No	1026 (63.2)	177 (52.4)^b^	849 (66.1)
Yes	586 (36.1)	158 (46.7)^b^	428 (33.3)
Not reported	11 (0.7)	3 (0.9)	8 (0.6)

a False discovery rate correction for multiple testing comparing positive findings patients with negative findings patients.

b *P* < 0.001.

c Provided responses included: Mexican, Dominican, Ashkenazi Jewish, and Middle Eastern.

**Table 2:** Positive genetic findings and diagnostic yield according to pretest clinical disease category

Clinical Disease Category	Full Cohort	Positive Cases		
	*N* (%)	No. of Cases	Proportion of all Positive Cases, %	Diagnostic Yield (per Disease), %
All Categories	1623	338	100	20.8
Cystic nephropathy	262 (16.1)	130	38.5	49.6
Nephropathy associated with hypertension	260 (16.0)	37	10.9	14.2
Proteinuric disease suggestive of a primary glomerulopathy	236 (14.5)	40	11.8	16.9
Nephropathy associated with diabetes mellitus	206 (12.7)	18	5.3	8.7
ESKD	190 (11.7)	28	8.3	14.7
CKD of unknown etiology	132 (8.1)	24	7.1	18.2
Nephrolithiasis with family history	58 (3.6)	5	1.5	8.6
Congenital nephropathy	50 (3.1)	10	3.0	20.0
Early onset, severe, or familial hypertension	46 (2.8)	5	1.5	10.9
Hematuria	45 (2.8)	15	4.4	33.3
Tubulointerstitial disease of unknown etiology	44 (2.7)	6	1.8	13.6
Electrolyte and/or acid base disorder	43 (2.6)	11	3.3	25.6
Thrombotic microangiopathy	4 (0.2)	0	0.0	0.0
Not reported	47 (2.9)	9	2.7	19.1

### Genetic Findings and Diagnostic Yield

**Figure 1:**
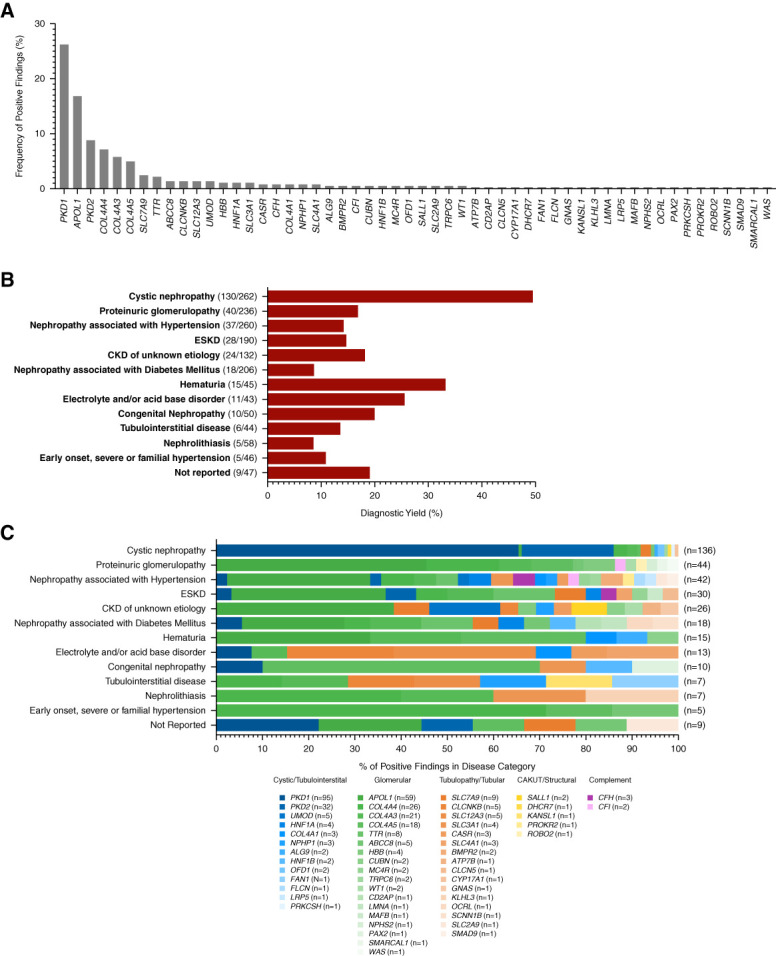
**Positive genetic findings according to pretest clinical diagnosis.** (A) The frequency of positive genetic findings across 54 genes identified in 338 patients. A total of 362 positive findings were identified, which included 317 patients with a single gene finding, 18 patients with two gene findings, and three patients with three gene findings. (B) Diagnostic yield of positive genetic findings within each clinical kidney disease categorization provided by physicians at enrollment. Diagnostic yield was calculated as the proportion of patients within each category with a positive genetic finding. Note: No positive findings were identified in patients categorized with thrombotic microangiopathy; as such, this category is not represented graphically in analyses pertaining to patients with positive findings. (C) The distribution of the 362 positive genetic findings across the clinical kidney disease categories. Genes are grouped by broad kidney disease types and ordered by prevalence among the cohort: cystic/tubulointerstitial (blue), glomerular (green), tubulopathy/tubular (orange), CAKUT/structural (yellow), complement (purple).

Among the 1623 patients tested, 338 patients had a positive result, including 61 *APOL1* high-risk genotypes, for an overall diagnostic yield of 20.8%. The median age among patients with a positive result was significantly younger than those with a negative result (48 versus 57 years, *P* < 0.001; Table 1). Over six percent (6.2%; *n*=21) of positive patients had findings in more than one gene, of which ten had an *APOL1* high-risk genotype (Supplemental Table 8). Positive findings spanned 54 genes and were most frequently identified in *PKD1* (26.2%), *APOL1* (16.9%), *PKD2* (8.8%), *COL4A4* (7.2%), *COL4A3* (5.8%), and *COL4A5* (5.0%) (Figure 1A). In total, 82% of positive findings were among patients in the six most common pretest clinical disease categories: cystic nephropathies (38.5%), proteinuric disease suggestive of a primary glomerulopathy (11.8%), nephropathies associated with hypertension (10.9%) or diabetes (5.3%), and ESKD (8.3%) and CKD of unknown etiology (7.1%).

The greatest diagnostic yield of positive genetic findings was within the cystic nephropathy category (49.6%) (Figure 1B and Table 2). Variants in *PKD1* and *PKD2* comprised 85.3% of the positive findings in this group, and other ciliopathy-associated genes (*ALG9*, *PRKCSH*, *OFD1*) accounted for an additional 3% (Figure 1C and Supplemental Table 9).

The diagnostic yield among proteinuric disease suggestive of a primary glomerulopathy was 16.9% (Figure 1B). Among the positive findings in this group, 95.5% (42/44) were in genes associated with a glomerular pathology. Of these, variants in *COL4A3/4/5* (associated with Alport syndrome spectrum) comprised 31.8% of positive cases, and *APOL1* high-risk genotypes comprised 45.5% (Figure 1C, Table 2, and Supplemental Table 9).

In this study, nephropathies associated with hypertension or diabetes mellitus were two common clinical presentations that were attributed to a patient's CKD. The diagnostic yields within these categories were 14.2% and 8.7%, respectively (Figure 1B and Table 2). Findings spanned 22 genes in the hypertension group and 13 genes in the diabetes group. In both groups, *APOL1* high-risk genotypes were the most common finding, 31% and 22.2%, respectively. In the diabetes group, only one case had a finding in a gene known to be a primary driver of diabetes-mediated nephropathy (*ABCC8*, associated with maturity-onset diabetes of the young [MODY]) (Figure 1C and Supplemental Table 9).

The diagnostic yields among patients with CKD of unknown etiology and ESKD were 18.2% and 14.7%, respectively (Figure 1B and Table 2). Together, the genetic findings in these groups spanned over 20 different genes, representing all kidney disease pathologies (Figure 1C and Supplemental Table 9).

### Effect of Genetic Testing on Clinical Diagnosis

**Figure 2:**
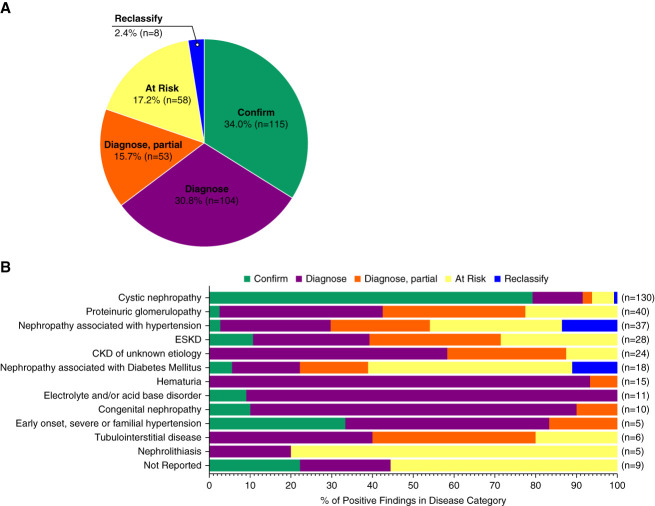
**Classification of clinical diagnosis with the application of genetic data.** Diagnostic utility of the molecular findings was assessed in the context of each patient's clinical diagnosis and medical history. (A) Breakdown of diagnostic effect of genetic findings among 338 positive patients. Confirm (*n*=115) 34.0%; diagnose (*n*=104) 30.8%; diagnose, partial (*n*=53) 15.7%; at risk (*n*=58) 17.2%; reclassify (*n*=8) 2.4%; of cases. Patients were designated at-risk if there were insufficient data provided to support classification into a diagnose or reclassify category. *APOL1* high-risk genotypes were categorized as *diagnose*, *partial*, or *at risk*. For patients with more than one positive finding, classification was assessed based on the presumed primary driver of disease. (B) Diagnostic utility classifications stratified by pretest clinical kidney disease categorization.

The effect of genetic findings on clinical diagnosis was analyzed for all 338 cases (Methods and Supplemental Methods). Genetic findings confirmed the clinical diagnosis for 34.0% of positive findings (*n*=115), of which 89.6% (103/115) were in patients with cystic nephropathies (79.2% of all positive cystic nephropathy cases) (Figure 2, A and B).

Genetic findings enabled a new diagnosis (including “diagnose” and “diagnose, partial”) that explained all or some of the patient's clinical phenotype in 46.4% (diagnose: *n*=104; diagnose, partial: *n*=53) of the 338 positive cases, respectively. For 17.2% (*n*=58) of patients, the genetic findings did not explain the clinical presentation; thus, these patients remained *at risk* for the development of features of the genetic condition. Genetic findings helped establish new diagnoses among a significant number of patients with hematuria (100% [15/15] of positives) and proteinuric glomerulopathy (75.0% [30/40] of positives), for which 80.0% and 31.8% resulted in establishing an Alport syndrome spectrum diagnosis, respectively. Among patients with CKD of unknown etiology and a positive finding, 87.5% (21/24) of cases were assigned a new diagnosis because of genetic testing, while the remaining 12.5% (3/24) remained at risk for the development of the genetic condition.

Eight cases (2.4%), in which the genetic finding differed from the patient's reported clinical diagnosis, were reclassified (Supplemental Table 10). Seven of the eight cases (87.5%) were originally categorized as nephropathy associated with hypertension (*n*=4), nephropathy associated with diabetes (*n*=2), or as early onset/familial hypertension (*n*=1). The remaining case (a positive finding in *PRKCSH*) was reclassified from a polycystic kidney disease to polycystic liver disease, which substantially altered the patient's prognosis (Figure 2B).

### Effect of Genetic Testing on Patient Clinical Management

**Figure 3:**
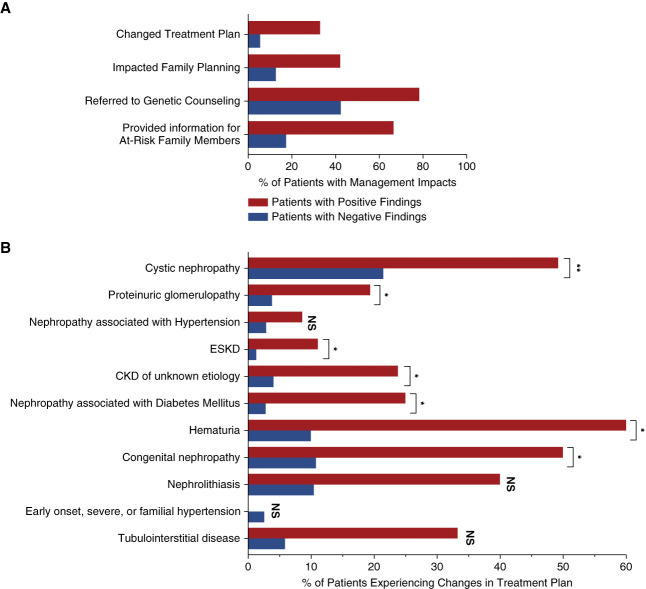
**The clinical effect of genetic diagnosis on patient management.** Physician responses to 1-month post-test questionnaires were used to assess the effect of genetic test findings on patient management. (A) Frequency of physician-reported changes because of genetic testing in four management categories (treatment plan changes, discussions around family planning, referrals to genetic counseling, and referrals for testing for at-risk family members) among patients with positive genetic findings (red) and negative genetic findings (blue) *P* < 0.001 for comparisons between positive and negative groups, for all categories. (B) Frequency of treatment plan changes among patients with positive genetic findings (red) and negative genetic findings (blue). *P < 0.05, **P < 0.001. NS, not significant.

Overall, it was reported that genetic testing resulted in changes to a patient management plan for 90.7% (284/313) of positive cases and 50.1% (597/1192) of negative cases (*P* < 0.001). One-month postgenetic testing, physicians reported that all four primary categories of patient management queried were affected in patients with positive genetic findings: changes in the treatment plan for 32.9% (103/313) of positive patients and 5.6% (67/1192) of negative patients; referrals to genetic counseling for 78.3% (245/313) of positive patients and 42.4% (506/1192) of negative patients; recommendations for family member referrals to genetic testing for 66.5% (208/313) of positive patients and 17.4% (207/1192) of negative patients; and in the initiation of discussions around family planning for 42.2% (132/313) of positive patients and 12.8% (152/1192) of negative patients (*P* < 0.001 for all comparisons; Figure 3A). Notably, for most patients with positive findings for which management changes were reported (75.4% [214/284]), more than one aspect management was reported to have been affected by genetic testing (one category: 24.6% [70/284]; two categories: 32.4% [92/284]; three categories: 19.0% [54/284]; four categories: 23.9% [68/284]).

In particular, changes in treatment plans were reported across the clinical kidney disease categories, ranging from 8.6% to 60.0% of patients with positive findings and 1.3%–21.5% of patients with negative findings. Patients with positive findings in four categories (cystic nephropathy, hematuria, congenital nephropathy, and nephrolithiasis with family history) reported changes in treatment in 40%–60% of cases (Figure 3B). Together these findings demonstrate that genetic testing affects the treatment and management of both patients with positive and negative genetic findings.

For ten patients with a positive genetic finding, it was reported that a kidney biopsy was not pursued because of the genetic result. In all of these cases, genetic testing provided a new or partial diagnosis (Supplemental Table 11). In addition, 196 patients enrolled in this study had a biopsy performed before genetic testing, suggesting that the prior biopsy result was either insufficient to fully clarify the patient's phenotype or prompted the genetic testing to better clarify the disease etiology. Among these patients, 19.4% (*n*=38) had a positive genetic finding, of which 71.1% (27/38) were able to receive a new diagnosis because of the findings (Supplemental Table 12).

## Discussion

RenaCARE is the first large-scale, real-world prospective study demonstrating the diagnostic and clinical utility of genetic testing in a geographically and ethnically diverse cohort of adults with CKD. Genetic testing with a broad, 385 kidney gene panel yielded positive findings in one fifth of adult patients with CKD, spanning 54 genes that encompassed a range of kidney disease pathologies. In almost half of patients with a positive genetic finding, the molecular diagnosis allowed for new genetically determined diagnoses and/or reclassification of a clinical diagnosis. The results of this interim analysis demonstrate that genetic findings improve the precision of a clinical diagnosis. Overall, genetic testing affected 90.7% of patients with positive genetic findings, and half of the patients with negative findings in areas of patient management including changes in treatment plan which were reported in a third of positive patients.

Outcomes in patients with CKD can be affected by genetic testing by enabling physicians to tailor management on the basis of accurate diagnoses. Studies have shown that genetic testing can reclassify a prior diagnosis in 11%–54% of patients with CKD and can establish an etiology in 12%–56% of patients with CKD/ESKD without a primary renal diagnosis.^4–10^ Many molecular diagnoses established in this study spanned disease categories, demonstrating the phenotypic ranges associated with the conditions. For instance, pathogenic variants in *COL4A3/4/5* were identified in patients within all 12 clinical diagnostic categories. Hence, as suggested in diagnostic guidelines for Alport syndrome,^22^ genetic testing may be more sensitive and specific than biopsy in diagnosing Alport syndrome and associated risk for progressive CKD and ophthalmologic and audiologic concerns. Furthermore, our findings indicate that genetic disorders are frequent in nephropathies attributed to either hypertension or diabetes mellitus.

Changes to patient management plans were reported in 90.7% of cases with positive genetic findings, confirming the putative implications previously reported by Groopman *et al.*^6^ The types of patient management queried in this study (changes to treatment plan, discussions around family planning, referrals for genetic counseling, and referrals for family testing) represent broad areas of patient care that each have varied implications for a given patient. It is important to not overlook the role of genetic counseling or patient education. This is important for the contextualization and personalized interpretation of test results, which can help patients understand their disease course and future care options.

Guidelines for the management of CKD specify certain lifestyle changes after a CKD diagnosis to avoid a “nephrotoxic lifestyle,” including management of BP, avoidance of nephrotoxic agents (e.g., non-steroidal anti-inflammatory drugs), dietary recommendations, and an active lifestyle.^23^ Several studies have demonstrated that genetic testing could lead to an earlier and more accurate diagnosis than standard-of-care practices,^22^ leading to improved patient outcomes. For example, knowledge of an *APOL1* high-risk genotype has been shown to lead to lifestyle changes that improved patient diet, exercise, and BP management, as well as eligibility for interventional trials.^24,25^ In addition, confirmation of diagnosis through genetic testing can lead to a projected course of disease and closer monitoring that can allow for initiation of treatment as early as possible. Treatments, such as renin–angiotensin–aldosterone system blockade in patients with Alport syndrome^22^ and tolvaptan in autosomal dominant polycystic kidney disease patients with *PKD1* or *PKD2* gene findings, can delay progression to kidney failure.^26,27^ Furthermore, positive genetic findings can help physicians confirm the need for such treatments. In addition, accurate diagnosis can prevent the use of ineffective or nephrotoxic treatments, as can occur in individuals with steroid-resistant nephrotic syndrome or FSGS, which often have a genetic etiology.^28^

Numerous practice guidelines have been issued recommending genetic testing as a first-line diagnostic tool for CKD.^14,22,29–32^ For example, guidelines for the management of Alport syndrome recommend genetic testing as the gold standard for diagnosis given the improved sensitivity and specificity over kidney biopsy.^33^ Furthermore, a molecular diagnosis allows for testing of at-risk family members, provides guidance about the eligibility of living-related kidney donors, and enables strategies for the management and surveillance of extrarenal complications.^22^ Similarly, the European Renal Association-European Dialysis and Transplant Association Working Group for Inherited kidney disease and the Taskforce of the European Rare Kidney Disease Reference Network recently issued recommendations for implementing genetic testing for patients with kidney disease to achieve an earlier and more accurate diagnosis that would avoid more invasive or costly diagnostic tests, potentially enable patients to receive targeted therapies and enroll in specific genetically stratified clinical trials.^32^ Ongoing long-term follow-up in the RenaCARE study aims to assess, with more granularity, the patient management changes observed after a genetic diagnosis.

Despite the acknowledged benefits of genetic testing on the management of CKD,^6,9^ barriers to the adoption of genetic testing for CKD exist, including insufficient education about recognizing genetic disease, identifying appropriate genetic tests, lack of access to genetic counseling and appropriate subspecialty referral, and concerns about insurability for patients.^12,13,34–36^ In addition, adoption of genetic diagnostics will require practitioners' acceptance that ongoing variant classification and gene discovery can lead to future changes in the interpretation of genetic results. Finally, a lack of familiarity and trust in genetic testing from physicians and patients can hinder implementation of changes on the basis of genetic findings.

This study had some limitations. First, only 385 genes were interrogated, leaving the possibility that additional untested genetic etiologies went undetected and that the 20.8% diagnostic yield measurement may be an underestimate. However, there are also drawbacks to the use of smaller phenotype-directed gene panels or whole-exome sequencing^37^; this 385 gene panel was designed to optimize the clinical benefits and costs.^15^ Second, physician responses regarding the aspects of clinical management were limited to options on the questionnaire. As such, details of specific treatments, extrarenal manifestation referrals, changes in disease prognosis, follow-up and outcomes of physician referrals, and the execution of these actions in practice may not have been captured; thus, these findings may not reflect all aspects of the overall utility. Given the intent of the RenaCARE study to understand the clinical utility of genetic testing in a real-world clinical setting across a variety of clinical kidney diagnosis, this study was designed to keep eligibility broad and to not specify the selection of specific patient types for enrollment. Third, as a single-armed, unblinded study, there is the potential for selection bias, the Hawthorne effect, and other confounding factors. However, owing to the highly heterogeneous nature of CKD and thus CKD-related genetic findings (*i.e.*, different variants are associated with different natural histories of disease), performance of a controlled study with matched study arms would be difficult to achieve. Furthermore, study designs, such as randomized control trials, may not have captured the information needed to assess the current state of genetic testing practices in nephrology. As such, these effects are unlikely to detract from the significance of the study findings and are informative to our understanding of current testing practices. Finally, this interim analysis was limited to physician responses 1 month after the results were returned. Longer follow-up of this cohort will provide a better long-term assessment of diagnosis and clinical care after genetic testing.

In summary, genetic testing with a 385 gene panel for CKD provided a high rate of new diagnoses or additional clarification of a clinical diagnosis. The improved diagnosis accuracy resulted in changes in management in a high proportion of patients with both positive and negative findings, including treatment plans, supporting incorporation of genetic testing into standard care for kidney disease patients.^14^

## Supplementary Material

## Data Availability

Partial restrictions to the data and/or materials apply. All data is included in the manuscript and/or supporting information.

## Supplemental Material

Supplemental Table 1. List of RenaCARE investigators and sites.

Supplemental Figure 1. Locations of RenaCARE sites.

Supplemental Figure 2. CONSORT diagram for RenaCARE trial.

Supplemental Table 2. Study inclusion and exclusion criteria. Collection of baseline information. Classification of genetic diagnosis.

Supplemental Table 3. Baseline clinical data and histories.

Supplemental Table 4. Pretest physician questionnaire.

Supplemental Table 5. Post-test physician questionnaire (1-month follow-up).

Supplemental Table 6. Patients with *IFT140* loss-of-function variants.

Supplemental Table 7. Patients with reclassification of VUS to P/LP positive genetic finding.

Supplemental Table 8. Cases with multiple positive findings.

Supplemental Table 9. Frequency of positive gene findings according to pretest clinical disease category.

Supplemental Table 10. Patients with reclassified diagnoses.

Supplemental Table 11. Patients for whom biopsy was not pursued based on genetic test results.

Supplemental Table 12. Patients with positive genetic findings that were biopsied before genetic testing.
