# Supplementary material for: The Clinical Utility of Genetic Testing in the Diagnosis and Management of Adults with Chronic Kidney Disease
Source: J Am Soc Nephrol. 2023 Oct 5;34(12):2039–50. doi: 10.1681/ASN.0000000000000249 (PMC10703084; doi:10.1681/ASN.0000000000000249)
Supplement: Supplementary file 1 [file jasn-34-2039-s001.pdf]

The Clinical Utility of Genetic Testing in the Diagnosis and Management of Adults With Chronic Kidney Disease

Neera Dahl, MD, PhD;[1] Michelle S. Bloom, PhD;[2] Fouad T. Chebib, MD;[3] Dinah Clark, MS CGC;[2] Maggie Westemeyer, MS CGC;[2] Sara Jandeska, MD;[2] Zhiji Zhang, MS;[2] Hila Milo Rasouly, PhD;[4] Victoria Kolupaeva, PhD;[4] Maddalena Marasa, MD;[4] Varshasb Broumand, MD;[5] Richard A Fatica, MD;[6] Dominic S Raj, MD;[7] Zachary P. Demko, PhD;[2] Kyle Marshall, MS;[2] Sumit Punj, PhD FACMG;[2] Hossein Tabriziani, MD;[2] Sangeeta Bhorade, MD;[2] Ali G. Gharavi, MD[4]

1. Division of Nephrology and Hypertension, Mayo Clinic, Rochester, MN, USA
2. Natera, Inc., Austin, TX, USA
3. Division of Nephrology and Hypertension, Mayo Clinic, Jacksonville, FL, USA
4. Division of Nephrology, Department of Medicine, Columbia University College of Physicians and Surgeons, New York, NY, USA
5. South Texas Renal Care Group, San Antonio, TX, USA
6. Department of Kidney Medicine, Cleveland Clinic, Cleveland, OH, USA
7. Division of Kidney Diseases and Hypertension, George Washington University School of Medicine and Health Sciences, Washington, DC, USA

Table of Contents

Table S1: List of RenaCARE Investigators and Sites ***2***
Figure S1: Locations of RenaCARE Sites ***3***
Figure S2. CONSORT Diagram for RenaCARE Trial. ***4***
Table S2: Study inclusion and exclusion criteria ***5***
Supplemental Methods ***6***
Collection of baseline information **6**
Classification of genetic diagnosis **6**
Table S3: Baseline clinical data and histories ***7***
Table S4: Pre-Test Physician Questionnaire ***8***
Table S5: Post-Test Physician Questionnaire (One Month Follow-Up) ***9***
Table S6: Patients with IFT140 loss-of-function variants ***12***
Table S7: Patients with reclassification of VUS to P/LP positive genetic finding ***12***
Table S8: Cases with Multiple Positive Findings ***13***
Table S9: Frequency of positive gene findings according to pre-test clinical disease category ***15***
Table S10: Patients with Reclassified Diagnoses ***17***
Table S11: Patients for whom biopsy was not pursued based on genetic test results ***18***
Table S12: Patients with Positive Genetic Findings that were Biopsied Prior to Genetic Testing ***19***

Table S1: List of RenaCARE Investigators and Sites

Investigator	Institution	City	State
Ali Gharavi, MD	Columbia University Irving Medical Center Dept of Medicine	New York	NY
Arman Faravardeh, MD, FACP	Balboa/California Institute of Renal Research (CIRR)	San Diego	CA
Syeda Ahmad, MD	University of Pittsburgh Medical Center (UPMC) - Department of Medicine Renal Electrolyte Division	Pittsburg	PA
Dominic Raj, MBBS, MD, DM, DNB, FASN, FACP	George Washington Medical Faculty Associates	Washington	DC
Simin Goral, MD	University of Pennsylvania - Renal, Electrolyte, and Hypertension Dept	Philadelphia	PA
Chebib Fouad, MD, FASN	Mayo (Rochester)	Rochester	MN
Neera Dahl, MD, PhD	Yale university	New Haven	CT
Maryam Gondal, MBBS, MD	Yale university	New Haven	CT
Reza Mizani, MD, FASN	Texas Kidney Care	San Antonio	TX
Richard Fatica, MD	Cleveland Clinic	Cleavland	OH
Melanie Barrido, MD, FASN	NANI (Fort Wayne)	Fort Wayne	IN
Robert Szewc, MD	Kidney & Hypertension Transplant Associates	San Antonio	TX
Harmeet Singh, MD, FASN, FACP	Western Nephrology and Metabolic Bone Disease, PC	Denver	CO
Suneel Udani, MD	NANI (Hinsdale)	Hinsdale	IL
Neville Dossabhoy, MD, FACP, FASN	University of MS Medical Center	Jackson	MS
Carl Dukes, MD, FACP, FASN	US Renal Care (USRC) - Houston St.	San Antonio	TX
Carolina Arias, MD	South Texas Renal Care Group (USRC)	San Antonio	TX
F. David Newby, MD, PhD	Nephrology & Hypertension Specialists, PC (USRC)	Dalton	GA
David Lefler, DO	Liberty Dialysis/US Renal Care (USRC)	Anchorage	AK
Varshi Broumand, MD, MHA, FACP, FASN	US Renal Care (USRC) - Westover Hills	San Antonio	TX
Ayodele Erinle, MD, FACP	USRC Kidney Research (USRC)	Gallup	NM
Dayan Gandhi, MD, MSc	Renal Consultants Medical Group (part of USRC)	Northridge	CA
Nelson Kopyt, DO, FASN, FNKF, FACP, CPI	Northeast Clinical Research Center	Bethlehem	PA

Vladamir Liberman, DO	PRINE Health	Rockville Center	NY
Melchiore Vernace, MD, FACP	Doylestown Hospital	Doyleston	PA
George Frem, MD, FACP, FASN	Renal Care Consultants P.C.	Johnstown	PA
Astha Gupta, MD	Einstein Medical Center	Philadelphia	PA
William Hoffman, MD	UPMC Pinnacle Harrisburg	Harrisburg	PA
Yeong-Hau Howard Lien, MD, PhD	AKDHC Medical Research Services, LLC (Arizona Kidney Disease & Hypertension Centers)	Bullhead City	AZ
Anup Patel, MD	Saint Barnabas Medical Center	Livingston	NJ
Syed Babar, MBBS, MD	Southeastern Clinical Research Institute, LLC	Augusta	GA
Naveed Masani, MD, FACP, FASN	NYU Langone Hospital—Long Island	Long Island	NY

Figure S1: Locations of RenaCARE Sites

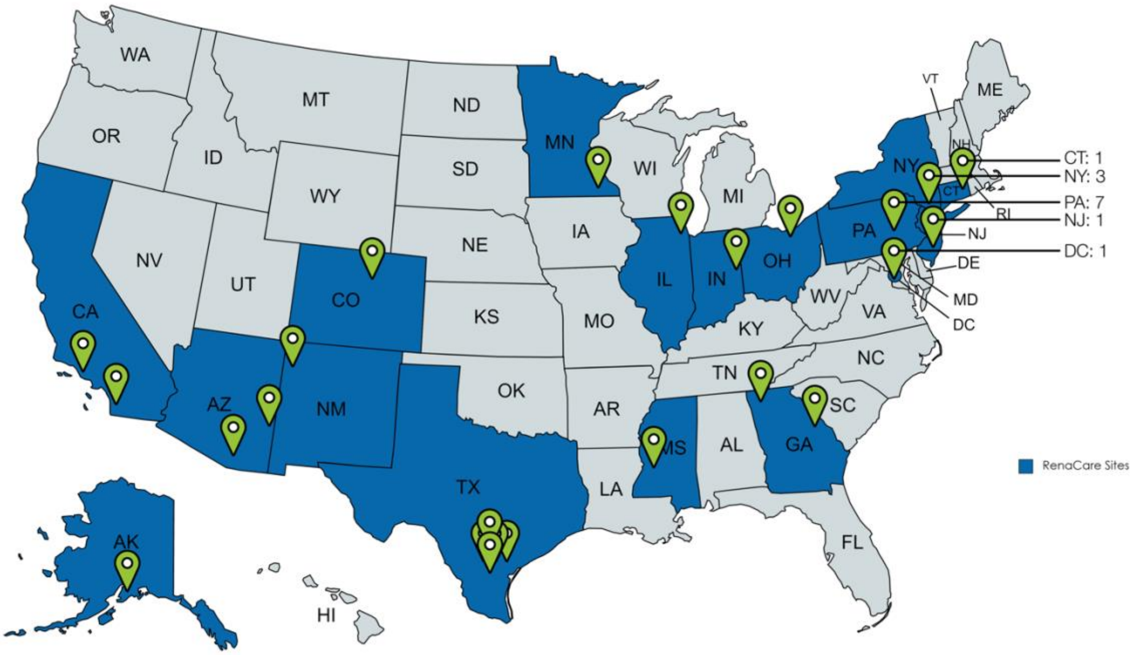

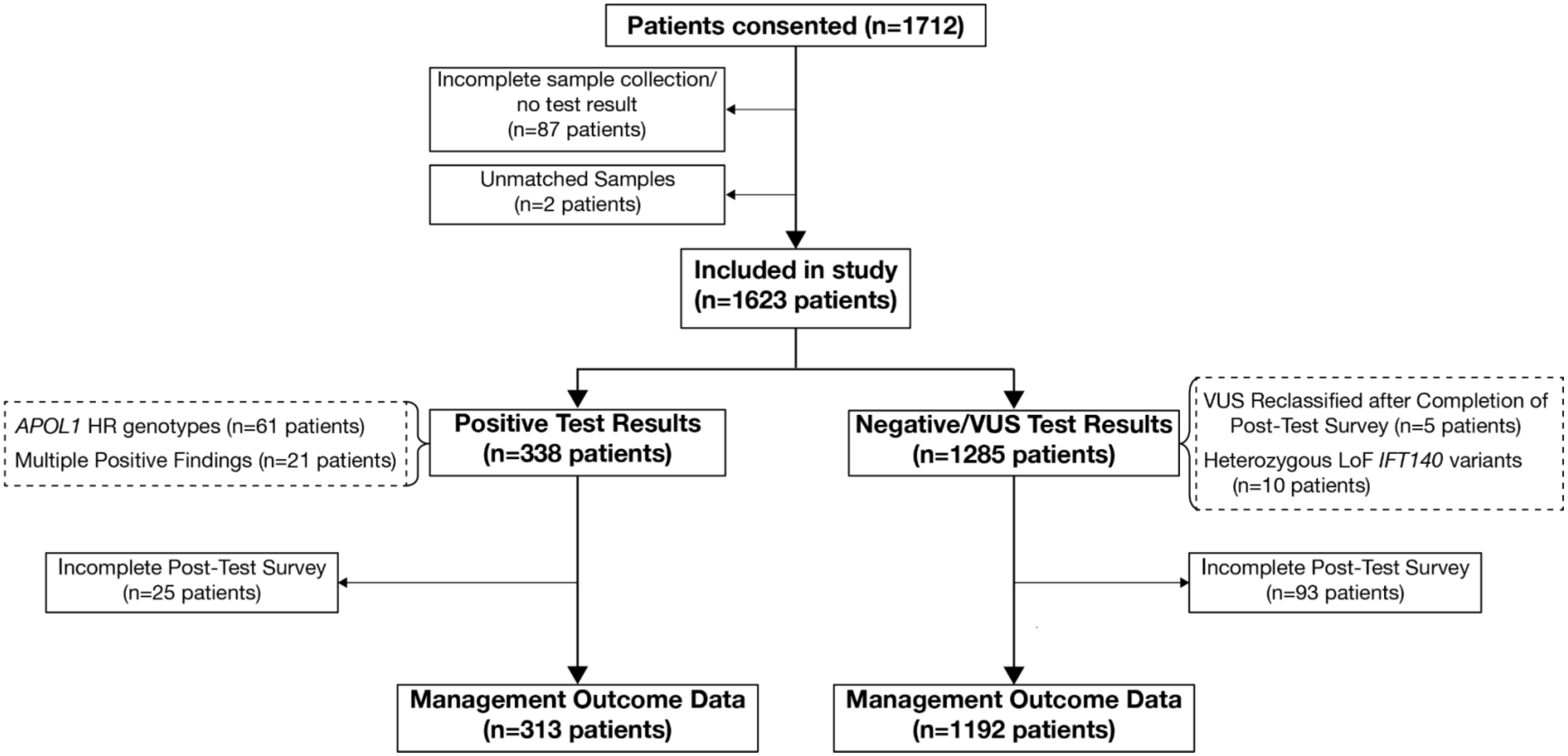

Figure S2. CONSORT Diagram for RenaCARE Trial.

CONSORT flow diagram shows the classification of patients according to positive or negative result. A total of 338 patients had positive genetic results, including 61 patients with high risk *APOL1* genotypes. Among the positive patients, 21 had more than one genetic finding, resulting in a total of 362 positive genetic findings. The diagram shows patients with completed management outcome data within each subgroup. These patients were used to evaluate clinical utility of genetic testing

Table S2: Study inclusion and exclusion criteria

Inclusion criteria
—--------------------

- ✔ 18 years and older at time of signing the informed consent
- ✔ For patients 65 years and older: absence of family history of CKD or suspicion of genetic etiology of kidney disease
- ✔ Diagnosis kidney disease, and/or one of the following:
  - Kidney disease not otherwise specified
  - Nephropathy associated with diabetes mellitus
  - Nephropathy associated with hypertension
  - Cystic nephropathy
  - Chronic kidney disease of unknown cause after standard nephrological evaluation
  - End stage renal disease
  - Congenital nephropathy
  - Tubulointerstitial disease of unknown etiology
  - Proteinuria disease suggestive of a primary glomerulopathy
  - Hematuria
  - Early onset, severe, or familial hypertension
  - Thrombotic microangiopathy
  - Electrolyte and/or acid-base disorder
  - Nephrolithiasis with family history
- ✔ Able to read, understand, provide written informed consent
- ✔ Willing and able to comply with the study-related procedures

Exclusion criteria
—------------------------

- ✔ History of kidney transplant
- ✔ Clinical features and kidney biopsy diagnosis strongly indicative of a secondary nephropathy (e.g., diabetic nephropathy, lupus nephritis, acute kidney injury)
- ✔ Previously confirmed diagnosis of a monogenic etiology of kidney disease from previous genetic testing
- ✔ Receipt of blood transfusion within 30 days of study blood draw

Supplemental Methods

Collection of baseline information

At the time of enrollment, physicians provided baseline (pre-test) information for each patient which included patient demographic data, clinical signs and symptoms, past medical history including medications, family history, laboratory data, imaging studies, and renal biopsy findings if available **(Table S2)**.

The physician pre-test questionnaire included ten multiple-choice questions and free text inquiring about the patient's clinical diagnosis; anticipated prognosis; treatment plan; consideration for biopsy; subspecialty referral; presence of extrarenal disease and the reason for genetic testing **(Table S3)**.

The physician post-test questionnaires documented additional diagnostic testing, changes to medications, referrals to clinical trials, evaluation of extrarenal disease, use of genetic counselors and subspecialists, plan for renal biopsy, and recommendation of at-risk family members to seek genetic testing **(Table S4)**.

Classification of genetic diagnosis

Adjudication of the patients clinical diagnosis (including both medical history/clinical information and clinical kidney disease categorization, if provided) with genetic findings:

1. A case was defined as ”confirm” when the condition associated with the genetic finding was consistent with the pre-test clinical diagnosis.

2. A case was defined as "diagnose" or "diagnose, partial" when the condition associated with the genetic finding was consistent (or partially consistent) with the reported clinical findings but not specified by the physician in the patient history.

3. A case was defined as ”reclassified” when the condition associated with the genetic finding was a new clinical diagnosis that differed from the pre-test clinical diagnosis.

4. A case was defined as "at-risk finding", when the patient did not appear to have signs or symptoms of the clinical diagnosis associated with the genetic finding but remained at risk for features of the genetic condition.

Cases with *APOL1* high-risk genotypes were considered only for the "diagnose, partial" or "at risk" categories. An APOL1 case was categorized as "diagnose, partial" if the patient's clinical findings were within the *APOL1* spectrum of disease or as "at-risk" if *APOL1* could not explain the patient's findings **(Table S9)**.

Cases with more than one positive finding were categorized based on the molecular finding that was presumed to be the primary driver of disease **(Table S7)**.

Table S3: Baseline clinical data and histories

Demographics
- Date of Birth:
- Premature birth? Yes No
- Birth Weight:
- Age:
- Sex at Birth: Male Female
- Is subject pregnant? Yes No
- Ethnicity:
- Birth Weight:
- Gestational Age: Weeks
- Race:

Family history
- Family history of CKD:
  - o Mother Father Sibling Child Other (free text)
- Family history of Hypertension:
  - o Mother Father Sibling Child Other (free text)
- Family history of Nephrolithiasis:
  - o Mother Father Sibling Child Other (free text)

Medications
- Within 30 days prior to sample collection
- clinically relevant changes in medication prescription/use at subsequent visit

Medical History
- Hematuria
- Allergic Disorders
- Psychiatric Disorders
- Proteinuria
- Congenital Anomalies of Kidney and/or Urinary Tract
- Polycystic or Cystic Nephropathy
- Kidneys of Small Size
- Lower Urinary Tract Obstruction
- Kidney Stones/Nephrolithiasis
- Electrolyte or Acid Base Disorder
- Recurrent/Frequent UTIs
- Exposure to Nephrotoxic Agents
- Thrombotic Microangiopathy
- Tubulointerstitial Disease
- Cardiovascular Disease
- Hypertension
- Peripheral Arterial Disease
- Stroke
- Autoimmune Disease
- Systemic Infections
- Thromboembolic Events
- Cancer
- Gastrointestinal Disease
- Lung Disease
- Neurological
- Sickle Cell Disease
- Diabetes Mellitus Type 1
- Diabetes Mellitus Type 2
- Gestational Diabetes
- Medication Related Diabetes
- Other (free text)

Vital Signs at Baseline Visit
- Collection Date:
- Heart Rate:
- Systolic Blood Pressure:
- Diastolic Blood Pressure:
- Height:
- Weight:

Laboratory Data
- GFR (measured by 24 hour creatinine clearance)
- GFR (measured by iothalamate clearance)
- Renal Function Panel (Na, K, Cl, HCO3, BUN, Cr, glucose, Ca, alb, phosphorous)
- 25OH & 1,25OH vitamin D
- Serum magnesium
- Intact PTH
- CBC
- Hemoglobin
- %HbA1c
- lipid panel (total cholesterol, HDL, LDL, triglycerides)
- B-type natriuretic peptide
- random urine protein & creatinine
- 24-hour urine protein and creatinine
- imaging studies ordered for kidney disease evaluation
- renal biopsy if performed as part of patient evaluation

Table S4: Pre-Test Physician Questionnaire

1. What is your patient’s clinical diagnosis (choose one)?
   Nephropathy with diabetes mellitus
   Nephropathy with hypertension
   Cystic nephropathy
   Congenital nephropathy
   Tubulointerstitial disease of unknown etiology
   Proteinuric disease suggestive of a primary glomerulopathy
   Hematuria
   Early onset, severe or familial hypertension
   Thrombotic microangiopathy
   Electrolyte and/or acid base disorder
   Nephrolithiasis with family history
   CKD of unknown cause after standard nephrological evaluation
   End stage kidney disease (ESRD)

2. What is the most likely prognosis for your patient in the next five years (choose one)?
   Remission of current active disease
   Possible relapsing of kidney disease
   Stable kidney function
   Slow progression to ESRD
   Rapid progression to ESRD

3. What is your treatment plan for this patient (choose all that apply)?
   Conservative therapy only (e.g. renin angiotensin blockade, lolled pressure control, diet)
   SGLT2 inhibition
   Immunosuppression with steroids
   Immunosuppression with other agents (e.g. calcineurin inhibitors, MMF, etc.)
   Refer for dialysis
   Refer for transplantation
   Refer for second opinion
   Refer to other subspecialist (i.e. Ophthalmologist,
   Otolaryngologist,...)
   for a clinical trial
   Other (free text)

4. Are you planning a kidney biopsy (choose one)?
   Yes, this patient has never had a biopsy
   Yes, even though he had biopsies in the past
   No, this patient has already had a diagnostic biopsy
   No

5. Is transplant recommended?
   Yes
   No

6. Would you consider a living related donor for this patient?
   Yes
   No
   I don't know

7. Does this patient have extra-renal health problems related or unrelated to the kidney disease?
    Yes
    No
    I don't know

8. Have you ever referred this patient to other sub-specialists?
    Yes
    No

9. Do you expect the genetic result to impact the management of kidney disease recommended to your patient?
    - Yes
    - No

10. What led you to consider genetic testing? (Check all that apply)
    - Strong family history of kidney disease
    - Early onset of kidney disease
    - Exceptionally severe or atypical presentation
    - Extra-renal features (e.g. structural congenital anomalies; dysmorphic features)
    - Clinical features suggestive of a specific genetic diagnosis
    - Kidney disease of unknown etiology
    - A genetic result can alter treatment plan
    - I am offering genetic testing to all my patients

Table S5: Post-Test Physician Questionnaire (One Month Follow-Up)

1. What is your patient's clinical diagnosis? (free text)

2. What was this Renasight test result? (check all that apply)
    - Negative
    - Positive
    - Carrier
    - Variant of Uncertain Significance (VUS)
    - Unable to analyze

3. Did the Renasight result provide a genetic diagnosis for your patient's kidney disease?
    - Yes
    - Partially (free text, specify)
    - No
    - I don't know

4. If a VUS was identified, what did you recommend to your patient?
    - To disregard this result
    - To follow-up in a year to learn whether the variant interpretation changed
    - To undergo clinical tests to help interpret this variant
    - To consider it as diagnostic
    - To discuss cascade testing in family members with genetic counselor
    - N/A: no VUS variant was identified
    - Other (free text)

5. If a carrier variant reported, what did you recommend to your patient?
    - To disregard this result

- To follow-up in a year to learn whether the variant interpretation changed
- To undergo additional genetic tests to help interpret this variant
- To consider it as diagnostic
- To discuss cascade testing in family members with genetic counselor
- N/A: no carrier variant was reported
- Other (free text)

6. If no genetic diagnosis was reported, did you recommend the following to your patient? (Check all that apply)
    - To consider that his/her kidney disease is not genetic
    - To discuss the opportunity to undergo additional genetic testing with a genetic counselor
    - To undergo a clinical whole-exome sequencing testing
    - To recommend genetic counseling to other family members with kidney disease
    - N/A: a genetic diagnosis was reported
    - Other (free text)

7. After receiving the Renasight result, did you refer your patient to genetic counseling?
    - Yes
    - No, he/she already talked with a genetic counselor about the result
    - No, I explained the result and do not see the need to refer to genetic counseling

8. Did the Renasight result impact your discussion about family planning with your patient?
    - Yes
    - No, the result didn't impact prior discussions on family planning
    - No, I haven't discussed family planning with my patient

9. What is your treatment plan for this patient?
    - Conservative therapy only (e.g. renin angiotensin blockade, lolled pressure control, diet)
    - SGLT2 inhibition
    - Immunosuppression with steroids
    - Immunosuppression with other agents (e.g. calcineurin inhibitors, MMF, etc.)
    - Refer for dialysis
    - Refer for transplantation
    - Refer for second opinion
    - Refer to other subspecialists (i.e. Ophthalmologist, Otolaryngologist,...)
    - Refer for a clinical trial
    - Other (free text)

10. Did the genetic result impact your decision to obtain a kidney biopsy?
    - Yes, I ordered a biopsy because of the genetic result
    - Yes, I did not order a biopsy because of the genetic result
    - No, I ordered a biopsy independently of the genetic result
    - No, I did not order a biopsy independently of the genetic result
    - This patient already has had a biopsy

11. Did your treatment plan change because of the genetic results?
    - Yes
    - No

12. What is the most likely prognosis for your patient in the next five years?
    - Remission of current active disease

- Possible relapsing of kidney disease
- Stable kidney function
- Slow progression to ESRD
- Rapid progression to ESRD

13. Transplant recommended?
    Yes
    No

14. Are you considering a living related donor?
    - Yes
    - No

15. Does this patient have extra-renal health problems related or unrelated to the kidney disease?
    Yes
    No

16. Did you recommend genetic testing for family members?
    Yes
    No

17. Did the experience with this patient change your interest in genetic testing? (Check all that apply)
    - Yes; I will discuss genetic testing with more patients
    - Yes; I will refer my patients to genetic counseling more often
    - Yes; I will order genetic testing more often
    - Yes; I will order genetic testing less often
    - No; it did not change my interest in referring my patients for genetic counseling
    - No; it did not change my interest in ordering genetic testing for my patients

Table S6: Patients with IFT140 loss-of-function variants

Clinical Kidney Disease Categorization (pre-test)	Gene	Variant
Cystic nephropathy	*IFT140*	c.2399+1G>T (p.?)
CKD of unknown cause after standard nephrological evaluation	*IFT140*	c.3219C>A (p.Tyr1073*)
Cystic nephropathy	*IFT140*	c.2399+1G>T (p.?)
Nephropathy associated with Hypertension	*IFT140*	c.1406_1430del (p.Leu469Glnfs*11)
Cystic nephropathy	*IFT140*	c.1010-1G>A (p.?)
Cystic nephropathy	*IFT140*	c.1377G>A (p.Trp459*)
Cystic nephropathy	*IFT140*	c.2980C>T (p.Gln994*)
Nephropathy associated with Diabetes Mellitus (DM)	*IFT140*	c.2578-2A>G (p.?)
Cystic nephropathy	*IFT140*	c.3160C>T (p.Gln1054*)
Cystic nephropathy	*IFT140*	c.2399+1G>T (p.?)

Table S7: Patients with reclassification of VUS to P/LP positive genetic finding

Clinical Kidney Disease Categorization (pre-test)	Gene	Variant
cystic nephropathy	*PKD2*	c.1349G>A
cystic nephropathy	*PKD1*	c.1543G>A
cystic nephropathy	*PKD1*	c.1543G>A
proteinuric disease suggestive of glomerulopathy	*RRM2B*	c.122G>A
proteinuric disease suggestive of glomerulopathy	*COL4A3*	C3546_3548dup

Table S8: Cases with Multiple Positive Findings

Genetic Findings	Pre-test Clinical Diagnosis	Diagnostic Impact Category
APOL1 (n=10)		
*ABCC8**<br>*APOL1*	Early onset, severe or familial hypertension	At risk
*COL4A4**<br>*APOL1*	Proteinuric disease suggestive of a primary glomerulopathy	diagnose
*COL4A3**<br>*COL4A4**<br>*APOL1*	Proteinuric disease suggestive of a primary glomerulopathy	diagnose
*PKD1**<br>*APOL1*<br>*CYP17A1*	Cystic nephropathy	confirm
*HBB**<br>*APOL1*	CKD of unknown cause after standard nephrological evaluation	diagnose
*HBB**<br>*APOL1*	Proteinuric disease suggestive of a primary glomerulopathy	confirm
*APOL1**<br>*MC4R*	ESKD	diagnose, partial
*APOL1**<br>*SLC3A1*<br>*TTR*	Nephropathy associated with Hypertension	diagnose, partial
*SMAD9**<br>*APOL1*	Nephropathy associated with Hypertension	diagnose
*APOL1**<br>*TTR*	Nephropathy associated with Hypertension	diagnose, partial
**Non-*APOL1*** (n=11)		
*CASR**<br>*SALL1*	CKD of unknown cause after standard nephrological evaluation	diagnose

*CFH** *TRPC6**	Nephropathy associated with Hypertension	at risk
COL4A3 *PKD1**	Cystic nephropathy	confirm
COL4A3 *SLC12A3**	Electrolyte and/or acid base disorder	diagnose
*COL4A4** *COL4A5**	Early onset, severe or familial hypertension	diagnose
*COL4A4** *KANSL1*	Tubulointerstitial disease of unknown etiology	diagnose
COL4A4 *PKD1**	Cystic nephropathy	confirm
*PKD1** *SLC7A9*	Cystic nephropathy	confirm
*PKD1** *TRPC6*	Cystic nephropathy	confirm
*PKD1** *TTR*	End stage kidney disease (ESRD)	confirm
PKD2 *SLC4A1*	Electrolyte and/or acid base disorder	diagnose

* gene with positive finding with the greatest impact on classification

Table S9: Frequency of positive gene findings according to pre-test clinical disease category

	Total Gene Findings	Cystic nephropathy	Proteinuric glomerulopathy	Nephropathy with Hypertension	End stage kidney disease	CKD of unknown etiology	Nephropathy with Diabetes	Hematuria	Electrolyte/acid base disorder	Congenital nephropathy	Familial hypertension	Tubulointerstitial disease	Nephrolithiasis	Not Reported
**Gene**	**N=362**	**N=136**	**N=44**	**N=42**	**N=30**	**N=26**	**N=18**	**N=15**	**N=13**	**N=10**	**N=7**	**N=7**	**N=5**	**N=9**
PKD1	95 (26.2)	89 (65.4)	0 (0.0)	1 (2.4)	1 (3.3)	0 (0.0)	1 (5.6)	0 (0.0)	0 (0.0)	1 (10.0)	0 (0.0)	0 (0.0)	0 (0.0)	2 (22.2)
APOL1	61 (16.9)	1 (0.7)	20 (45.5)	13 (31.0)	10 (33.3)	6 (23.1)	4 (22.2)	0 (0.0)	0 (0.0)	0 (0.0)	4 (57.1)	0 (0.0)	1 (20.0)	2 (22.2)
PKD2	32 (8.8)	27 (19.9)	0 (0.0)	1 (2.4)	2 (6.7)	0 (0.0)	0 (0.0)	0 (0.0)	1 (7.7)	0 (0.0)	0 (0.0)	0 (0.0)	0 (0.0)	1 (11.1)
COL4A4	26 (7.2)	4 (2.9)	7 (15.9)	3 (7.1)	2 (6.7)	1 (3.8)	1 (5.6)	5 (33.3)	0 (0.0)	0 (0.0)	1 (14.3)	1 (14.3)	1 (20.0)	0 (0.0)
COL4A3	21 (5.8)	3 (2.2)	3 (6.8)	2 (4.8)	3 (10.0)	1 (3.8)	2 (11.1)	3 (20.0)	1 (7.7)	1 (10.0)	0 (0.0)	1 (14.3)	1 (20.0)	0 (0.0)
COL4A5	18 (5.0)	1 (0.7)	4 (9.1)	0 (0.0)	0 (0.0)	2 (7.7)	0 (0.0)	4 (26.7)	0 (0.0)	5 (50.0)	1 (14.3)	0 (0.0)	0 (0.0)	1 (11.1)
SLC7A9	9 (2.5)	3 (2.2)	0 (0.0)	0 (0.0)	2 (6.7)	2 (7.7)	1 (5.6)	0 (0.0)	0 (0.0)	0 (0.0)	0 (0.0)	0 (0.0)	0 (0.0)	1 (11.1)
TTR	8 (2.2)	0 (0.0)	0 (0.0)	2 (4.8)	4 (13.3)	0 (0.0)	2 (11.1)	0 (0.0)	0 (0.0)	0 (0.0)	0 (0.0)	0 (0.0)	0 (0.0)	0 (0.0)
ABCC8	5 (1.4)	1 (0.7)	1 (2.3)	0 (0.0)	0 (0.0)	0 (0.0)	1 (5.6)	0 (0.0)	0 (0.0)	0 (0.0)	1 (14.3)	0 (0.0)	0 (0.0)	1 (11.1)
CLCNKB	5 (1.4)	0 (0.0)	0 (0.0)	0 (0.0)	0 (0.0)	1 (3.8)	0 (0.0)	0 (0.0)	3 (23.1)	0 (0.0)	0 (0.0)	1 (14.3)	0 (0.0)	0 (0.0)
SLC12A3	5 (1.4)	0 (0.0)	0 (0.0)	0 (0.0)	0 (0.0)	0 (0.0)	0 (0.0)	0 (0.0)	4 (30.8)	0 (0.0)	0 (0.0)	1 (14.3)	0 (0.0)	0 (0.0)
UMOD	5 (1.4)	0 (0.0)	0 (0.0)	1 (2.4)	0 (0.0)	4 (15.4)	0 (0.0)	0 (0.0)	0 (0.0)	0 (0.0)	0 (0.0)	0 (0.0)	0 (0.0)	0 (0.0)
HBB	4 (1.1)	0 (0.0)	3 (6.8)	0 (0.0)	0 (0.0)	1 (3.8)	0 (0.0)	0 (0.0)	0 (0.0)	0 (0.0)	0 (0.0)	0 (0.0)	0 (0.0)	0 (0.0)
HNF1A	4 (1.1)	0 (0.0)	0 (0.0)	2 (4.8)	1 (3.3)	0 (0.0)	1 (5.6)	0 (0.0)	0 (0.0)	0 (0.0)	0 (0.0)	0 (0.0)	0 (0.0)	0 (0.0)
SLC3A1	4 (1.1)	0 (0.0)	0 (0.0)	2 (4.8)	0 (0.0)	0 (0.0)	0 (0.0)	0 (0.0)	0 (0.0)	1 (10.0)	0 (0.0)	0 (0.0)	1 (20.0)	0 (0.0)
CASR	3 (0.8)	0 (0.0)	0 (0.0)	1 (2.4)	0 (0.0)	1 (3.8)	0 (0.0)	0 (0.0)	1 (7.7)	0 (0.0)	0 (0.0)	0 (0.0)	0 (0.0)	0 (0.0)
CFH	3 (0.8)	0 (0.0)	0 (0.0)	2 (4.8)	1 (3.3)	0 (0.0)	0 (0.0)	0 (0.0)	0 (0.0)	0 (0.0)	0 (0.0)	0 (0.0)	0 (0.0)	0 (0.0)
COL4A1	3 (0.8)	0 (0.0)	0 (0.0)	1 (2.4)	0 (0.0)	0 (0.0)	0 (0.0)	1 (6.7)	1 (7.7)	0 (0.0)	0 (0.0)	0 (0.0)	0 (0.0)	0 (0.0)
NPHP1	3 (0.8)	0 (0.0)	0 (0.0)	1 (2.4)	0 (0.0)	1 (3.8)	0 (0.0)	0 (0.0)	0 (0.0)	0 (0.0)	0 (0.0)	1 (14.3)	0 (0.0)	0 (0.0)
SLC4A1	3 (0.8)	0 (0.0)	0 (0.0)	0 (0.0)	1 (3.3)	0 (0.0)	0 (0.0)	0 (0.0)	2 (15.4)	0 (0.0)	0 (0.0)	0 (0.0)	0 (0.0)	0 (0.0)
ALG9	2 (0.6)	1 (0.7)	0 (0.0)	0 (0.0)	0 (0.0)	0 (0.0)	0 (0.0)	1 (6.7)	0 (0.0)	0 (0.0)	0 (0.0)	0 (0.0)	0 (0.0)	0 (0.0)
BMPR2	2 (0.6)	0 (0.0)	0 (0.0)	2 (4.8)	0 (0.0)	0 (0.0)	0 (0.0)	0 (0.0)	0 (0.0)	0 (0.0)	0 (0.0)	0 (0.0)	0 (0.0)	0 (0.0)
CFI	2 (0.6)	0 (0.0)	1 (2.3)	1 (2.4)	0 (0.0)	0 (0.0)	0 (0.0)	0 (0.0)	0 (0.0)	0 (0.0)	0 (0.0)	0 (0.0)	0 (0.0)	0 (0.0)
CUBN	2 (0.6)	0 (0.0)	0 (0.0)	0 (0.0)	0 (0.0)	1 (3.8)	0 (0.0)	1 (6.7)	0 (0.0)	0 (0.0)	0 (0.0)	0 (0.0)	0 (0.0)	0 (0.0)
HNF1B	2 (0.6)	0 (0.0)	0 (0.0)	0 (0.0)	0 (0.0)	0 (0.0)	1 (5.6)	0 (0.0)	0 (0.0)	1 (10.0)	0 (0.0)	0 (0.0)	0 (0.0)	0 (0.0)
MC4R	2 (0.6)	0 (0.0)	0 (0.0)	1 (2.4)	1 (3.3)	0 (0.0)	0 (0.0)	0 (0.0)	0 (0.0)	0 (0.0)	0 (0.0)	0 (0.0)	0 (0.0)	0 (0.0)
OFD1	2 (0.6)	2 (1.5)	0 (0.0)	0 (0.0)	0 (0.0)	0 (0.0)	0 (0.0)	0 (0.0)	0 (0.0)	0 (0.0)	0 (0.0)	0 (0.0)	0 (0.0)	0 (0.0)

	Total Gene Findings	Cystic nephropathy	Proteinuric glomerulopathy	Nephropathy with Hypertension	End stage kidney disease	CKD of unknown etiology	Nephropathy with Diabetes	Hematuria	Electrolyte/acid base disorder	Congenital nephropathy	Familial hypertension	Tubulointerstitial disease	Nephrolithiasis	Not Reported
SALL1	2 (0.6)	0 (0.0)	0 (0.0)	0 (0.0)	0 (0.0)	2 (7.7)	0 (0.0)	0 (0.0)	0 (0.0)	0 (0.0)	0 (0.0)	0 (0.0)	0 (0.0)	0 (0.0)
SLC2A9	2 (0.6)	0 (0.0)	0 (0.0)	1 (2.4)	0 (0.0)	0 (0.0)	0 (0.0)	0 (0.0)	0 (0.0)	0 (0.0)	0 (0.0)	0 (0.0)	0 (0.0)	1 (11.1)
TRPC6	2 (0.6)	1 (0.7)	0 (0.0)	1 (2.4)	0 (0.0)	0 (0.0)	0 (0.0)	0 (0.0)	0 (0.0)	0 (0.0)	0 (0.0)	0 (0.0)	0 (0.0)	0 (0.0)
WT1	2 (0.6)	0 (0.0)	1 (2.3)	0 (0.0)	0 (0.0)	1 (3.8)	0 (0.0)	0 (0.0)	0 (0.0)	0 (0.0)	0 (0.0)	0 (0.0)	0 (0.0)	0 (0.0)
ATP7B	1 (0.3)	0 (0.0)	0 (0.0)	0 (0.0)	0 (0.0)	1 (3.8)	0 (0.0)	0 (0.0)	0 (0.0)	0 (0.0)	0 (0.0)	0 (0.0)	0 (0.0)	0 (0.0)
CD2AP	1 (0.3)	0 (0.0)	0 (0.0)	0 (0.0)	0 (0.0)	0 (0.0)	1 (5.6)	0 (0.0)	0 (0.0)	0 (0.0)	0 (0.0)	0 (0.0)	0 (0.0)	0 (0.0)
CLCN5	1 (0.3)	0 (0.0)	0 (0.0)	0 (0.0)	1 (3.3)	0 (0.0)	0 (0.0)	0 (0.0)	0 (0.0)	0 (0.0)	0 (0.0)	0 (0.0)	0 (0.0)	0 (0.0)
CYP17A1	1 (0.3)	1 (0.7)	0 (0.0)	0 (0.0)	0 (0.0)	0 (0.0)	0 (0.0)	0 (0.0)	0 (0.0)	0 (0.0)	0 (0.0)	0 (0.0)	0 (0.0)	0 (0.0)
DHCR7	1 (0.3)	1 (0.7)	0 (0.0)	0 (0.0)	0 (0.0)	0 (0.0)	0 (0.0)	0 (0.0)	0 (0.0)	0 (0.0)	0 (0.0)	0 (0.0)	0 (0.0)	0 (0.0)
FAN1	1 (0.3)	0 (0.0)	0 (0.0)	0 (0.0)	0 (0.0)	0 (0.0)	0 (0.0)	0 (0.0)	0 (0.0)	0 (0.0)	0 (0.0)	1 (14.3)	0 (0.0)	0 (0.0)
FLCN	1 (0.3)	0 (0.0)	0 (0.0)	1 (2.4)	0 (0.0)	0 (0.0)	0 (0.0)	0 (0.0)	0 (0.0)	0 (0.0)	0 (0.0)	0 (0.0)	0 (0.0)	0 (0.0)
GNAS	1 (0.3)	0 (0.0)	0 (0.0)	0 (0.0)	0 (0.0)	1 (3.8)	0 (0.0)	0 (0.0)	0 (0.0)	0 (0.0)	0 (0.0)	0 (0.0)	0 (0.0)	0 (0.0)
KANSL1	1 (0.3)	0 (0.0)	0 (0.0)	0 (0.0)	0 (0.0)	0 (0.0)	0 (0.0)	0 (0.0)	0 (0.0)	0 (0.0)	0 (0.0)	1 (14.3)	0 (0.0)	0 (0.0)
KLHL3	1 (0.3)	0 (0.0)	0 (0.0)	0 (0.0)	0 (0.0)	0 (0.0)	0 (0.0)	0 (0.0)	0 (0.0)	0 (0.0)	0 (0.0)	0 (0.0)	1 (20.0)	0 (0.0)
LMNA	1 (0.3)	0 (0.0)	0 (0.0)	0 (0.0)	0 (0.0)	0 (0.0)	1 (5.6)	0 (0.0)	0 (0.0)	0 (0.0)	0 (0.0)	0 (0.0)	0 (0.0)	0 (0.0)
LRP5	1 (0.3)	0 (0.0)	0 (0.0)	1 (2.4)	0 (0.0)	0 (0.0)	0 (0.0)	0 (0.0)	0 (0.0)	0 (0.0)	0 (0.0)	0 (0.0)	0 (0.0)	0 (0.0)
MAFB	1 (0.3)	0 (0.0)	0 (0.0)	0 (0.0)	1 (3.3)	0 (0.0)	0 (0.0)	0 (0.0)	0 (0.0)	0 (0.0)	0 (0.0)	0 (0.0)	0 (0.0)	0 (0.0)
NPHS2	1 (0.3)	0 (0.0)	1 (2.3)	0 (0.0)	0 (0.0)	0 (0.0)	0 (0.0)	0 (0.0)	0 (0.0)	0 (0.0)	0 (0.0)	0 (0.0)	0 (0.0)	0 (0.0)
OCRL	1 (0.3)	0 (0.0)	0 (0.0)	0 (0.0)	0 (0.0)	0 (0.0)	1 (5.6)	0 (0.0)	0 (0.0)	0 (0.0)	0 (0.0)	0 (0.0)	0 (0.0)	0 (0.0)
PAX2	1 (0.3)	0 (0.0)	0 (0.0)	0 (0.0)	0 (0.0)	0 (0.0)	0 (0.0)	0 (0.0)	0 (0.0)	1 (10.0)	0 (0.0)	0 (0.0)	0 (0.0)	0 (0.0)
PRKCSH	1 (0.3)	1 (0.7)	0 (0.0)	0 (0.0)	0 (0.0)	0 (0.0)	0 (0.0)	0 (0.0)	0 (0.0)	0 (0.0)	0 (0.0)	0 (0.0)	0 (0.0)	0 (0.0)
PROKR2	1 (0.3)	0 (0.0)	0 (0.0)	1 (2.4)	0 (0.0)	0 (0.0)	0 (0.0)	0 (0.0)	0 (0.0)	0 (0.0)	0 (0.0)	0 (0.0)	0 (0.0)	0 (0.0)
ROBO2	1 (0.3)	0 (0.0)	1 (2.3)	0 (0.0)	0 (0.0)	0 (0.0)	0 (0.0)	0 (0.0)	0 (0.0)	0 (0.0)	0 (0.0)	0 (0.0)	0 (0.0)	0 (0.0)
SCNN1B	1 (0.3)	0 (0.0)	0 (0.0)	0 (0.0)	0 (0.0)	0 (0.0)	1 (5.6)	0 (0.0)	0 (0.0)	0 (0.0)	0 (0.0)	0 (0.0)	0 (0.0)	0 (0.0)
SMAD9	1 (0.3)	0 (0.0)	0 (0.0)	1 (2.4)	0 (0.0)	0 (0.0)	0 (0.0)	0 (0.0)	0 (0.0)	0 (0.0)	0 (0.0)	0 (0.0)	0 (0.0)	0 (0.0)
SMARCAL1	1 (0.3)	0 (0.0)	1 (2.3)	0 (0.0)	0 (0.0)	0 (0.0)	0 (0.0)	0 (0.0)	0 (0.0)	0 (0.0)	0 (0.0)	0 (0.0)	0 (0.0)	0 (0.0)
WAS	1 (0.3)	0 (0.0)	1 (2.3)	0 (0.0)	0 (0.0)	0 (0.0)	0 (0.0)	0 (0.0)	0 (0.0)	0 (0.0)	0 (0.0)	0 (0.0)	0 (0.0)	0 (0.0)

Table S10: Patients with Reclassified Diagnoses

Gene/ Associated Condition	Pre Test Clinical Information	Rationale for Reclassification
PRKCSH<br>Polycystic Liver Disease 1	Cystic nephropathy; ADPKD	ADPLD
COL4A3<br>Alport Syndrome	Nephropathy associated with Diabetes Mellitus (DM); Proteinuria, hematuria, hypertension, DMT2, CKD3	Renal features can be explained by Alport Syndrome
COL4A4<br>Alport Syndrome	Nephropathy associated with Diabetes Mellitus (DM); Moderate hematuria, proteinuria, DMT2	Renal features can be explained by Alport Syndrome
COL4A3<br>Alport Syndrome	Nephropathy associated with Hypertension; proteinuria, hypertension	Renal features can be explained by Alport Syndrome
HNF1A<br>Maturity-Onset Diabetes of the Young	Nephropathy associated with Hypertension; Diabetes (diagnosed at age 8)	Diabetes etiology in genetic finding
HNF1A<br>Maturity-Onset Diabetes of the Young	Nephropathy associated with Hypertension; Diabetes (diagnosed at age 29)	Diabetes etiology in genetic finding
NPHP1<br>Juvenile nephronophthisis/ Senior-Loken syndrome/Joubert syndrome	Nephropathy associated with Hypertension; Hypertension onset at age 12	Hypertension and echogenic kidneys are features of NPHP1-related disease
UMOD<br>Autosomal Dominant Tubulointerstitial Kidney Disease (ADTKD)	Nephropathy associated with Hypertension, small right kidney, hyperlipidemia, hypertension, CKD4	Clinical features can be explained by UMOD-related ADTKD

Table S11: Patients for whom biopsy was not pursued based on genetic test results

Disease Category	Genetic Diagnosis	Diagnostic Impact	Clinical Data
Nephropathy with HTN	*APOL1*	At risk	47yo African-American male, proteinuria, fam hx HTN; SCr 1.9mg/dl
Nephropathy with HTN	*APOL1*	at risk	62yo African-American female with HTN, strong fam hx kidney disease; SCr 1.02mg/dl; bilateral solitary cysts
Nephropathy with HTN	*NPHP1*	reclassify	33yo White male with pediatric onset HTN; SCr 3.4mg/dl
Nephropathy with HTN	*APOL1/ SLC3A1/ TTR*	diagnose, partial	48yo male with HTN
Nephropathy with DM	*APOL1*	At risk	40yo African-American male with DM x6yrs, eGFR 58ml/min; proteinuria, HTN, obesity; HbA1c 12.3%
Hematuria	*COL4A4*	diagnose	70yo White male, microscopic hematuria, SCr 1.39mg/dl
Hematuria	*COL4A4*	diagnose	57yo White female with microscopic hematuria, proteinuria, hypertension
Hematuria	*COL4A5*	diagnose	54yo White female with microscopic hematuria, HTN, eGFR 78ml/min
Hematuria	*COL5A5*	diagnose	22yo white male
congenital nephropathy	*COL4A5*	diagnose	55yo White female with hematuria, proteinuria

Table S12: Patients with Positive Genetic Findings that were Biopsied Prior to Genetic Testing

Clinical diagnosis	Genetic Diagnosis	Diagnostic Impact	Histologic finding
Nephropathy associated with Hypertension	*APOL1*	Diagnose, partial	Limited kidney biopsy composed predominantly of medulla with extensive chronic changes.
Nephropathy associated with Hypertension	*APOL1*	Diagnose, partial	DNP
Nephropathy associated with Hypertension	*APOL1*	At risk molecular finding	Acute tubular injury, diffuse mild arterionephrosclerosis
Proteinuric disease suggestive of a primary glomerulopathy	*APOL1*	At risk molecular finding	DNP
Proteinuric disease suggestive of a primary glomerulopathy	*APOL1*	At risk molecular finding	Membranous nephropathy
Proteinuric disease suggestive of a primary glomerulopathy	*APOL1*	At risk molecular finding	Diffuse segmental and global sclerosing glomerulopathy, moderate, with glomerulomegaly
Proteinuric disease suggestive of a primary glomerulopathy	*APOL1*	At risk molecular finding	Membranous nephropathy
Proteinuric disease suggestive of a primary glomerulopathy	*APOL1*	At risk molecular finding	Diffuse mesangial and mild focal segmental membranoproliferative and sclerosing glomerulonephritis with monoclonal IgG3-kappa deposits

Proteinuric disease suggestive of a primary glomerulopathy	*APOL1*	At risk molecular finding	Chronic thrombotic microangiopathy, arteriosclerosis
Proteinuric disease suggestive of a primary glomerulopathy	*APOL1*	Diagnose, partial	DNP
Proteinuric disease suggestive of a primary glomerulopathy	*APOL1*	Diagnose, partial	Collapsing FSGS
Proteinuric disease suggestive of a primary glomerulopathy	*APOL1*	Diagnose, partial	FSGS
Proteinuric disease suggestive of a primary glomerulopathy	*APOL1*	Diagnose, partial	FS/FG mild glomerulosclerosis with glomerulomegaly and focal collapsing features; moderately severe arteriolosclerosis
Proteinuric disease suggestive of a primary glomerulopathy	*APOL1*	Diagnose, partial	FSGS
Proteinuric disease suggestive of a primary glomerulopathy	*APOL1*	Diagnose, partial	Focal segmental and global sclerosing glomerulopathy with mesangial electron dense deposits Arterio- and arteriolosclerosis, moderate.
Proteinuric disease suggestive of a primary glomerulopathy	*APOL1*	Diagnose, partial	FSGS
Proteinuric disease suggestive of a primary glomerulopathy	*ABCC8*	At risk finding	FSGS
Proteinuric disease suggestive of a	*APOL1*<br>*COL4A3**	Diagnose	DNP

primary glomerulopathy	*COL4A4**		
Proteinuric disease suggestive of a primary glomerulopathy	*APOL1*<br>*COL4A4**	Diagnose	DNP
Proteinuric disease suggestive of a primary glomerulopathy	*APOL1*<br>*HBB**	Confirm	DNP
Proteinuric disease suggestive of a primary glomerulopathy	*CFI*	At risk molecular finding	DNP
Proteinuric disease suggestive of a primary glomerulopathy	*COL4A3*	Diagnose, partial	DNP
Proteinuric disease suggestive of a primary glomerulopathy	*COL4A3*	Diagnose, partial	DNP
Proteinuric disease suggestive of a primary glomerulopathy	*COL4A4*	Diagnose, partial	IgAN
Proteinuric disease suggestive of a primary glomerulopathy	*COL4A4*	Diagnose	Focal mesangial proliferative glomerulonephritis, consistent with IgA nephropathy, with mild glomerulomegaly, segmental GBM thinning, and focal GBM textural irregularities. Tubular atrophy and interstitial fibrosis, mild, with scattered interstitial foam cells.
Hematuria	*COL4A4*	Diagnose	Thin basement membrane nephropathy
Proteinuric disease suggestive of a primary glomerulopathy	*COL4A5*	Diagnose	FSGS, mild mesangial immune complex deposition

Proteinuric disease suggestive of a primary glomerulopathy	*COL4A5*	Diagnose	irregular GBM, focal glomerular fibrinoid necrosis, FSGS
Congenital Nephropathy	*COL4A5*	Diagnose	DNP
Proteinuric disease suggestive of a primary glomerulopathy	*HBB*	Diagnose	Membranous glomerulopathy, stage 2-3, with segmental sclerosing features. Tubular atrophy & interstitial fibrosis, mild to moderate.
Proteinuric disease suggestive of a primary glomerulopathy	*NPHS2*	Diagnose	FSGS NOS but with thin GBMs noted on EM (mean 174.7 nm), raising suspicion for a type IV collagenopathy.
Proteinuric disease suggestive of a primary glomerulopathy	*ROBO2*	At risk molecular finding	Membranous nephropathy
Proteinuric disease suggestive of a primary glomerulopathy	*WAS*	Diagnose	IgAN
Hematuria	*CUBN*	Diagnose	normal size, normocellular mesangium, GBMs appear thin and delicate
Tubulointerstitial disease	*FAN1*	Diagnose	DNP
Tubulointerstitial disease	*NPHP1*	Diagnose	Consistent with chronic tubulointerstitial nephropathy, moderate
CKD of Unknown Etiology	*CUBN*	Diagnose, partial	DNP
Early, Severe, or Familial Onset Hypertension	*COL4A4*<br>*COL4A5*	Diagnose	DNP

DNP= data not provided; classification based on clinical data provided
